# A Role for Calcineurin in Alzheimer’s Disease

**DOI:** 10.2174/157015911798376316

**Published:** 2011-12

**Authors:** Lindsay C Reese, Giulio Taglialatela

**Affiliations:** Department of Neuroscience & Cell Biology, University of Texas Medical Branch at Galveston, Texas, 77555-1043, USA

**Keywords:** Alzheimers, amyloid beta, calcineurin, calcium.

## Abstract

Alzheimer’s disease (AD) is an incurable age-related neurodegenerative disorder characterized by profound memory dysfunction. This bellwether symptom suggests involvement of the hippocampus -- a brain region responsible for memory formation -- and coincidentally an area heavily burdened by hyperphosphorylated tau and neuritic plaques of amyloid beta (Aβ). Recent evidence suggests that pre-fibrillar soluble Aβ underlies an early, progressive loss of synapses that is a hallmark of AD. One of the downstream effects of soluble Aβ aggregates is the activation of the phosphatase calcineurin (CaN). This review details the evidence of CaN hyperactivity in ‘normal’ aging, models of AD, and actual disease pathogenesis; elaborates on how this could manifest as memory impairment, neuroinflammation, hyperphosphorylated tau, and neuronal death.

## 1 INTRODUCTION

Calcineurin (CaN), also known as protein phosphatase 2B (PP2B), is a calcium (Ca^2+^)-sensitive serine/threonine phosphatase highly expressed in the central nervous system (CNS) and originally isolated from mammalian brain [1]. Structurally, CaN is a heteromeric protein comprised of a catalytic subunit (CaNA) and a regulatory subunit (CaNB) [2]. While the catalytic site of CaNA is similar to protein phosphatase-1 (PP1) and protein phosphatase-2A (PP2A), the regulatory CaNB shares 30-50% sequence homology with calmodulin (CaM) [3]. Among this family of related phosphatases CaN is singular in that it is directly activated by CaM, making it uniquely and exquisitely responsive to Ca^2+ ^fluctuation [4]. Relatively unaffected by traditional phosphatase inhibitors, CaN is sensitive to the immuno-suppressants cyclosporine A and FK506 when they are bound to their respective immunophilins cyclophilin A and FKBP12 [5]. The use of these compounds, coupled with antisense RNA technology and transgenic models have only recently allowed investigation into the roles of CaN in cellular signaling. 

The designation “calcineurin” reflects the initial belief that it was uniquely expressed in neurons, where it is present in such high levels as to comprise 1% of total neural protein [2]. In fact, this important phosphatase -- conserved from yeast to man -- is present in a diverse group of cell types where it responds to the binding of activated CaM in multiple ways, including modulating immune responses [6], influencing muscle formation and remodeling [7], neuronal plasticity [8-9], and cell death [10-11]. Due to the promiscuous tendencies of the phosphatase, Ca^2+^ entry and subsequent CaN activation signals powerful cellular processes impacting cell survival and growth. Included on the list of CaN substrates are the phosphorylated forms of nuclear factor of activated T- cells (NFAT) [12]; cAMP response element binding (CREB) [8]; PP1 [8, 13]; microtubule-associated protein tau [14-15]; glycogen synthase kinase-3 beta (GSK-3β) [16, 17]; and Bcl-2 associated death protein (BAD) [10, 11]. The effects of CaN hyperactivity on these downstream proteins and the evidence for their involvement in AD pathogenesis will be the focus of this review.

## 2 CALCINEURIN AND SYNAPTIC FUNCTION

This section will briefly delineate the intimate relationship between CaN signaling and neuronal excitability [rev. in 3, 9]. CaN is able to dephosphorylate a number of ion channels, including L-type Ca^2+^ channels [18], potassium (K^+^) channels [19], and voltage-gated sodium (Na^+^) channels [20-21]. Through its impact on ion channels, CaN activation impacts the basal excitability of neurons.

CaN also exerts powerful effects on synaptic transmission in both the pre- and post-synaptic compartments. For instance, CaN has been reported to affect the release of neuro-transmitters from the pre-synaptic compartment by negatively regulating exocytotic release of glutamate. This outcome is likely due to the ability of CaN to dephosphorylate synapsin I, a phosphoprotein that tethers neurotransmitter-containing vesicles to the cytoskeleton [22]. When phosphorylated, the synapsin-associated vesicles are moved from the reserve vesicle pool to those ready to be released [23]. Therefore, CaN activation could theoretically reduce the amount of neurotransmitter released into the synapse. In addition, CaN signaling controls the speed of synaptic endocytosis, *via *its effect on the dephosphorylation of endocytotic proteins [24].

The phosphatase is also involved in post-synaptic events. CaN has been demonstrated to dephosphorylate the *N*-methyl-D-aspartate receptor (NMDA-R), which serves as a signal for the receptor gate to close. In this way, CaN activity desensitizes the receptors, resulting in less mean open time and lower open channel probability [25, 26]. CaN is known to enhance or prolong the desensitization period of other ligand-gated channels, including γ-aminobutyric acid (GABA) [27, 28], serotonin [29], and acetylcholine receptors [30].

Thus, CaN is intimately involved in a number of pathways that modulate neuronal excitability and synaptic activity. Dysregulation of this complex system would likely have far-reaching consequences on synaptic connectivity.

## 3 CALCINEURIN AND MECHANISMS OF MEMORY 

Given the initial impairments of memory, cognition, and concentration that mark early stage AD, this section will introduce the normal role of CaN in the physiological processes of memory. Long-term potentiation (LTP) is widely held to be the molecular basis of learning and memory [31]. The phenomenon of LTP was first described in the live rabbit hippocampus, where a brief tetanic burst of artificial electrical stimuli applied to the perforant pathway resulted in enhanced hippocampal neuronal transmission when a single shock was administered hours later [32]. Although the rabbit brain was not actually encoding memories during these sessions, long-lasting potentiation following repetitive neuronal firing was quickly identified as a putative mechanism of the learning process. LTP can be divided into “early” and “late” stages, the first of which involves the phosphorylation or membrane insertion of the existing pool of proteins [33], and the latter requires gene transcription and synthesis of new proteins [34].

One piece of cellular machinery necessary for both stages of LTP expression was determined to be the NMDA-R. Hippocampal pathways are enriched with these specialty ion channels, which become operational after an initial membrane depolarization sufficient to remove the magnesium block from the pore that allows the entry of Ca^2+^. In this way, NMDA-Rs act as coincidence detectors, opening only in response to bursts of synaptic transmission that exceed baseline neuronal firing. This allows for Ca^2+^entry that is activity and temporally specific, occurring as rapid pulses in the microsecond to millisecond range [35]. Subsequent Ca^2+^ binding to CaM results in the activation of downstream kinases and phosphatases, including Ca^2+^/calmodulin-dependent protein kinases (CaMKs) and CaN. Parallel phosphorylation pathways lead to the activation of CREB; pCREB is able to translocate to the nucleus and transcribe genes that produce proteins necessary for synaptic maintenance and formation [8].

A proposed allosteric model suggests that CaM is more likely to activate either CaN or CaMKs depending on local Ca^2+^ concentration [36]. Intense yet confined and transient Ca^2+^ increases – such as those through NMDA-Rs following tetanic stimulation – initially results in the preferential activation of CaMKII within the dendritic spines. However, as Ca^2+^ decreases, but before it returns to baseline, CaM is more likely to bind and activate CaN [36]. *Via *dephosphorylation of the dopamine- and c-AMP regulated phosphoprotein of Mr 32,000 (DARPP-32), CaN activates PP1 dephosphorylative capabilities, thus indirectly promoting PP1-dependent CREB dephosphorylation (inactivation) [37, 38]. PP1 also dephosphorylates CaMKII, consequently decreasing its kinase activity towards ion channels until the next wave of Ca^2+^ entry [13]. Such a setup allows for bidirectional synaptic plasticity, where a shift in Ca^2+^ levels serves as the switch between synapse growth (positive) and synapse pruning (negative). The latter is likely accomplished by long-term depression (LTD), an activity-dependent decrease in synaptic efficacy that is typically induced by a more modest but protracted rise in Ca^2+^ [39, 40]. LTD is facilitated through CaN activity and is thought to allow for more efficient neural storage.

The precise role of CaN in learning and memory has been probed by overexpressing, inhibiting, and knocking down the phosphatase. Targeted overexpression of CaN in the forebrain of mice impaired the transition from short-term to long-term memory as well as an intermediate form of LTP [41, 42]. Conditional genetic knockout of CaN in mouse forebrain resulted in impairment within a specific subset of hippocampal-dependent tasks including working and episodic memory [43]. However, knockdown of CaN expression with antisense oligonucleotides results in the facilitation of LTP and improved performance in the Morris water maze [44]. Similarly, partial CaN inhibition with a tunable, inducible rtTA system also facilitated LTP and performance in a hippocampal-dependent behavioral test [45]. Collectively, these studies suggest that equilibrium between positive and negative plasticity is critical for proper cognitive function; and that an appropriate level of CaN activity is central to this balance.

## 4 CALCINEURIN AND AGING

It is well recognized that aging has a profound effect on the welfare of neurons in the central nervous system (CNS). As part of a larger network not easily reproduced, post-mitotic CNS neurons are remarkably long-lived in comparison to other cell types such as epithelia. Only in the past forty years was it acknowledged that neurogenesis occurs in the adult brain, most of the neurons do not actively divide past puberty. An exception is the dentate gyrus of the hippocampus, where neurogenesis continues post-natally into adulthood [46, 47]. Although there is continued proliferation in certain brain regions, the average neuron in the aged brain has been exposed to decades of oxidative insults.

The brain is further disadvantaged by its high-energy metabolism and low endogenous anti-oxidant defenses [48]. Aging exacerbates these unfavorable conditions: evidence of increased oxidative stress and reduced mitochondrial function have been extensively documented in the aged brain [49]. Both of these factors decrease cellular ability to tightly regulate Ca^2+^ Fig. (**1**). Perturbation in Ca^2+^ levels are particularly troublesome for neurons, as numerous cellular processes are governed either by intracellular Ca^2+^ or downstream kinases and phosphatases, including LTP and LTD. Indeed, strict governance of Ca^2+^ is especially critical at the synapses of hippocampal pathways. These connections are borne out of intense, local Ca^2+ ^entry into dendritic spines containing high concentrations of NMDA-Rs. Positive and negative regulators of plasticity, including CaN, are also enriched within hippocampal neurons. Therefore, these networks responsible for learning and memory are particularly vulnerable to functional alterations with increasing age.

For twenty-five years it has been known that neurons from the aged brain have altered Ca^2+^ currents in comparison to young [50], possibly due to increased numbers of voltage-gated Ca^2+^ channels (VGCCs) [51]. More recent evidence suggests that Ca^2+^ entry through VGCCs augments a secondary Ca^2+^ flux from the endoplasmic reticulum to the cytosol through ryanodine receptors, and that this occurs to a greater degree in aged hippocampal neurons [52]. Regardless of the mechanism of Ca^2+^ increase, CaN activity is upregulated in the aging hippocampus. Impaired performance in the Morris water maze mirrors elevated CaN activity and cytosolic CaN expression in the aged rat hippocampus. These increases are paralleled by enhanced dephosphorylation of CaN-substrates BAD and CREB, as well as augmented activity of PP1 [53]. Electrophysiological studies suggest that LTD predominates over LTP in the aged rat brain, but that this is antagonized by CaN inhibition [54]. CaN may also exacerbate the already dysregulated Ca^2+^ homeostasis in aged brain; CaN modulates the activity of VGCCs in hippocampal cultures and inhibition with FK506 blocks the activity of these channels [55]. New studies in partially dissociated hippocampal “zipper” slices from young, middle-aged, and old rats show that this phenomenon also occurs *in vivo*, and suggests that CaN may directly activate VGCCs. Viral-mediated delivery of activated CaN to primary hippocampal neurons increased VGCC activity [56]. These results are attributable to enhanced “neuronal CaN activity.” However, immunohistochemical analyses of aged murine hippocampi show intense CaN staining in activated astrocytes [57], providing a possible role for CaN hyperactivity in triggering astrogliosis and inflammatory pathways.

## 5 CALCINEURIN AND Aβ

This section will discuss how certain species Aβ are able to hyperactivate CaN in models of AD. Data gathered from *in vitro*, *ex vivo*, and *in vivo* studies illustrates how dephosphorylation of CaN substrates can impact gene transcription, cell death, ion channel activity, and synaptic integrity.

The original “Amyloid Hypothesis” predicted that altered processing or clearance of Aβ resulted in plaque deposition and AD symptoms, but this proposal has undergone a revision in recent years. Although insoluble, fibrillar Aβ was initially believed to be central to disease pathogenesis, the latest evidence has indicated that soluble oligomeric Aβ is behind the earliest cognitive deficits [58]. Indeed, the proposition that small Aβ aggregates are able to affect cognition *via *synaptotoxic action has received robust experimental confirmation in the last decade. Oligomeric species have been shown to preferentially accumulate at the synapse in cultured hippocampal neurons [59, 60], where they are able to alter the shape, size, and protein composition of the dendritic spines [61]. Why oligomers are attracted to the synapse is not well understood, but several hypotheses have been suggested, such as influence of metal ion concentration [62]. 

Aβ disrupts the functionality as well as the structure of the synapse. Application of oligomeric Aβ counteracts the increase in AMPA phosphorylation that normally occurs following tetanic stimulation of rat hippocampal slices, precluding the expression of early LTP [63]. Synthetic Aβ inhibits late phase LTP in a CaN-dependent fashion during electrophysiological recordings [64, 65]. Soluble oligomeric Aβ from several sources (synthetic, cell culture, human brain extracts) facilitates electrically evoked LTD and causes a 33% reduction of dendritic spine density in organotypic hippocampal cultures. Both outcomes were preventable by the CaN inhibitor FK506 [66]. Collectively, these studies hint that Aβ-mediated activation of CaN promotes LTD over LTP. As discussed in previous sections, this important balance between positive and negative plasticity is already perturbed in the normal aged brain. Further exacerbation by oligomeric Aβ could putatively explain the pathological synaptic loss believed to underlie the early symptoms of AD.

The effect of Aβ on CaN activity can be explained by its ability to perturb intracellular Ca^2+^ Fig. (**1**). Certain aggregate species are able to act as Ca^2+^ channels in synthetic bilayer membranes [67], and Aβ is hypothesized to interact with several membrane receptors, including NMDARs [68], alpha-7 nicotinic receptors [69], or meta-botropic glutamate receptors [70]. Given its unknown function and cell surface receptor-like structure, it is possible that oligomers bind to and signal through full-length amyloid precursor protein (APP) [71]. Live Ca^2+^ imaging of SY5Y human neuroblastomas demonstrates that oligomeric Aβ is the only species that appreciably augments the concentration of cytosolic Ca^2+^ by disrupting the cellular membrane. This increase was reduced but not abolished when the experiment was performed in Ca^2+^ free conditions, with 30% of the rise coming from internal stores [72]. Whatever the source, these studies suggest that only oligomers should be capable of upregulating CaN activity, *via *the Ca^2+^ increase. Indeed, only oligomers raise intracellular Ca^2+^, CaN hyperactivity, and CaN dependent cell death in cell cultures [73, 74]. 

Multiphoton Ca^2+^ imaging of AD mouse models has revealed the extent of Ca^2+^ dysregulation provoked by Aβ *in vivo*. In aged double transgenic mice (APP/PS1) with cortical plaques, 20% of the neurites contained elevated resting Ca^2+ ^levels, much greater than the young double mutants and significantly higher than the 5% increase in aged wild-type mice or single mutants. Furthermore, Ca^2+ ^overload correlated with the proximity to Aβ plaques. An observation coincident with increased resting Ca^2+ ^was neuritic “beading” – a morphological change indicative of neuronal stress. This effect was partially attenuated by systemic treatment with FK506, again indicating that structural changes downstream of Ca^2+ ^overload are mediated in part by CaN [75]. Indeed it was later found that Aβ induces loss of dendritic spines, simplification of dendritic arborization and neuritic dystrophies through a CaN/NFAT-dependent mechanism [76].

CaN hyperactivity alters the ability of neurons to turn on protein synthesis that normally occurs during late-stage LTP. *In vitro* experiments have shown that pCREB levels as well as its transcriptional activity are diminished in a CaN-dependent fashion following treatment with oligomeric Aβ. The same study reported that hippocampal pCREB immuno-reactivity is reduced in the Tg2576 murine model of AD, but is restored by treatment with FK506 [74]. This animal model produces high levels of Aβ and first displays behavioral impairments at five months of age, coincident with the onset of elevated CaN activity [77]. Acute inhibition of CaN by FK506 improved the performance of these animals on a hippocampal-dependent fear conditioning paradigm [77] and novel object recognition as well [78]. Wild-type mice given a single intracerebroventricular injection of oligomeric Aβ exhibited similar deficits in the fear conditioning paradigm, again this was reversible with FK506 [65]. Together, these studies suggest that some of the behavioral impairments in AD mouse models could be explained by CaN hyperactivity and its subsequent effects on pCREB and synaptic plasticity.

The viability of neurons is also affected by Aβ, with CaN playing a central role. Application of Aβ has long been known to induce apoptosis in neuronal cultures [79]. This stringently controlled process is distinct from necrosis and necessitates the involvement of cellular signaling. One such pathway that leads to an apoptotic outcome is the CaN-mediated dephosphorylation of pBAD. Dephosphorylated BAD is able to dissociate from scaffolding proteins and translocate to the mitochondria, where it forms pro-apoptotic dimers with the protein Bcl-X(L), triggering cytochrome c release, thus initiating programmed cell death [10]. In SY5Y human neuroblastoma cells treated with increased concentrations of oligomeric Aβ there is a dose-dependent decrease in pBAD levels [74]. Treatment of primary cortical neurons with synthetic Aβ peptides increases CaN activity; reduces the level of phosphorylated BAD; and increases the amount of BAD found in the mitochondria [80]. These effects on cortical neurons were attenuated by the CaN inhibitor FK506, suggesting that some of the neurodegeneration seen in AD may be due to the ability of CaN to induce apoptosis *via *BAD.

As mentioned previously, CaN also dephosphorylates NFAT, allowing its translocation to the nucleus where it promotes the transcription of genes involved in cytokine production and inflammation [12]. Application of oligomeric Aβ increases NFAT activation in primary rat astrocyte cultures. This treatment also causes a significant reduction in excitatory amino acid transporter 2 (EAAT2) protein levels in astrocyte cultures, theoretically leaving extracellular glutamate levels high and increasing the likelihood of excitotoxic cell death. Inhibition of NFAT prevented Aβ-mediated elevation in glutamate and cell death [81]. 

The lines of evidence described above conceptually link Aβ-mediated Ca^2+ ^dysregulation, CaN hyperactivation, decreased synaptic plasticity, cell death, and neuro-inflammation. However, the models tested against the CaN hyperactivity hypothesis with relatively high levels of aggregated recombinant Aβ (the longer and stickier 42-amino-acid form), genetic overexpression of mutated APP prone to the generation of Aβ, and aberrant versions of APP processing enzymes termed presenilins (PS1 and PS2) isolated from a few kindreds with rare forms of familial AD. While this research has shed considerable light on possible mechanisms of Aβ synaptotoxicity and neuronal death, it is important to examine evidence that does not rely exclusively on atypical mutations of APP or presenilins, as sporadic, late-onset AD accounts for more than 90% of all cases with risk increasing dramatically with age. 

## 6 CALCINEURIN AND TAU

In addition to Aβ plaques, hyperphosphorylated tau is the other pathological signature of AD [82]. Tau is the major microtubule-associated protein (MAP), and is responsible for modulating the assembly and organization of microtubules, which facilitate intraneuronal transport [83]. Phosphorylation of tau decreases its affinity for the microtubules and the subsequent disassociation of tau increases the rate of microtubule depolymerization [84-85]. Thus, aberrant tau phosphorylation results in a loss of endogenous tau functions, as well as a toxic gain of function, where pathological tau assembled into paired-helical filaments (PHFs) sequesters normal tau [rev. in 86]. 

The hyperphosphorylated tau tangles that overwhelm the AD brain were conjectured to be the result of an imbalance between the kinases and phosphatases that interact with tau [86]. CaN was one of the phosphatases demonstrated to dephosphorylate tau, *in vitro* [14-15, 87]. At the same time, two independent groups reported that tangle-containing neurons and neurons surrounding plaques in AD brain showed strong CaN immunoreactivity [88, 89]; promoting the hypothesis that CaN modulated tau phosphorylation and suggested that decreased CaN activity may be in part responsible for hyperphosphorylation of tau [87]. Calcipressin, an endogenous CaN inhibitor, is found at high levels in AD brain, seemingly supporting the hypothesis that CaN phosphatase activity is decreased [90, 91]. For these reasons CaN was originally theorized to be down-regulated during AD pathogenesis, and that one of the downstream consequences was the hyperphosphorylation of tau. However, more recent evidence suggests that this view may be erroneous.

It has been demonstrated that both the A and B subunits of CaN associate directly with tau [92]. However, the binding of CaM to CaN impairs the binding between CaN and tau. These results suggest that CaN interacts with tau in basal Ca^2+^ conditions. So, when intracellular Ca^2+^ rises, activated CaM binds to CaN, disrupting its interaction with tau, and CaN’s ability to dephosphorylate tau is impaired. In such a scenario, an increase in Ca^2+^ hyperactivates CaN, which would paradoxically result in a decrease in CaN-mediated desphosphorylation of tau Fig. (**1**). CaN is also able to dephosphorylate glycogen synthase kinase-3 beta (GSK-3β) [16]. This activates GSK-3β, which is then able to phosphorylate tau at the same epitopes that are phosphorylated in AD brain [93].

Relatively few current studies have examined the interplay of tau and CaN. As it has become more evident that CaN activity is increased, researchers have began to re-explore the topic. For example, evidence from hippocampal slice cultures demonstrates that dendritic spine pathology requires CaN activation and downstream GSK-3β activation but amyloid beta-induced and tau-mediated neurodegeneration occuring in the cell soma occurs independently of CaN [94]. It is likely that additional research using current model systems will further clarify the relationship between CaN and tau in AD.

## 7 CALCINEURIN AND AD

This section will describe evidence for CaN hyper-activation within the actual disease state. Despite a growing amount of information from *in vitro*, *ex vivo*, and animal models, the evidence for dysregulation of CaN in actual AD brain is still scarce and inconclusive. Although sample sizes are much smaller, results from studies of human tissue from early and late-onset AD corroborate with those obtained from disease models to strengthen the case for CaN hyper-activation as a central factor in AD pathogenesis in both sporadic and familial AD pathogenesis. 

Recent studies suggest that CaN is in fact upregulated in AD brain. One small study on a small cohort (n = 7) of AD cerebral cortex found that in comparison to control brain (n = 5), overall phosphatase activity was decreased in AD. Conversely, nickel-stimulated increase of CaN activity was significantly higher in the prefrontal, but not sensorimotor cortex [95]. An investigation into CaN-mediated dephosphorylation of phosphorylated tau in human frontal cortex yielded no significant difference between AD and control brains [96]. However, this particular study employed Huntington disease samples as ‘control’. Such activity assays are only possible in rapidly autopsied tissue due to the swift oxidation of CaN post-mortem. Unfortunately similar experiments have not been done in the hippocampus, to-date. 

Incubation of brain extract with calpains produces CaN that is cleaved at lysine residue 501 as measured by mass spectometry. This 57-kDa cleaved version of CaN is elevated in AD homogenates from medial-temporal cortex. The truncated form maintains the autoinhibitory region, and thus is still dependent on CaM to be activated. In the presence of CaM, *in vitro* phosphatase activity is enhanced following cleavage [97]. A recent publication reported a 2 fold increase in the level of a 54-kDa fragment of CaN in the nuclear fraction of AD cortex [76]. These results were in disagreement with a publication from 2007, which found *decreased *immunoreactivity for CaN in an immuno-histochemical examination of AD frontal cortex. However, this study relied on a very small number of AD tissue samples (n = 3). Furthermore, when modeling the proposed Aβ-mediated CaN decrease in primary neuron lines, a very high level of Aβ (10μM) was necessary to obtain a significant decrease in CaN [98]. This concentration is physiologically irrelevant, and further studies are necessary to confirm the reported findings. 

Downstream of CaN, certain isoforms of NFAT (NFAT1 and 3) are increased in the nuclear fraction from AD hippocampal homogenate. These correlate with levels of soluble Aβ as well as Mini-Mental State Exam scores (MMSE), a standard measure of cognitive function [82]. Analysis of CREB and pCREB levels in human tissue show that amounts of pCREB are significantly lower in the AD hippocampus [99]. While this publication did not investigate the possible involvement of CaN, this report of decreased pCREB is circumstantial evidence that fits within the schematic of CaN-mediated cognitive dysfunction in AD. 

## 8 CONCLUSIONS

The greatest risk factor for developing AD is increasing age. While this is the case for many neurodegenerative conditions and may be a confounded correlation, it does suggest that something about aging neurons renders them especially susceptible to the ravages of AD. In older organisms, the brain is less plastic, in part due to a dysregulation of Ca^2+^ dynamics. The environment of the aged brain, further insulted by the presence of oligomeric Aβ, may result in an enhancement of CaN activity sufficient to explicate several negative outcomes observable in AD brain: decreased neurotransmission, synaptic loss, tau pathology, neuroinflammation, and cell death Fig. (**1**).

Therefore, it is prudent to consider the possibility of CaN inhibition as a pharmacological target in the development of novel AD therapies. FK506 and cyclosporine cause the unfortunate side-effect of immunosuppression *via *NFAT-mediated down-regulation of interleukin-2. This is an undesirable circumstance for aged patients already contending with compromised immune function. Uncompetitive NMDA-R antagonists, including memantine, have showed some promise at delaying the clinical progression of AD but not preventing the outcome [100]. Based on the evidence described here, the positive effects following such pharmacological regimens may be due to some prevention of Ca^2+^ entry. More efficacious therapies may require a different approach to preventing excessive intracellular Ca^2+^, regulation of CaM signaling, or perhaps a more precise inhibitor of neuronal CaN. 

## Figures and Tables

**Fig. (1):**
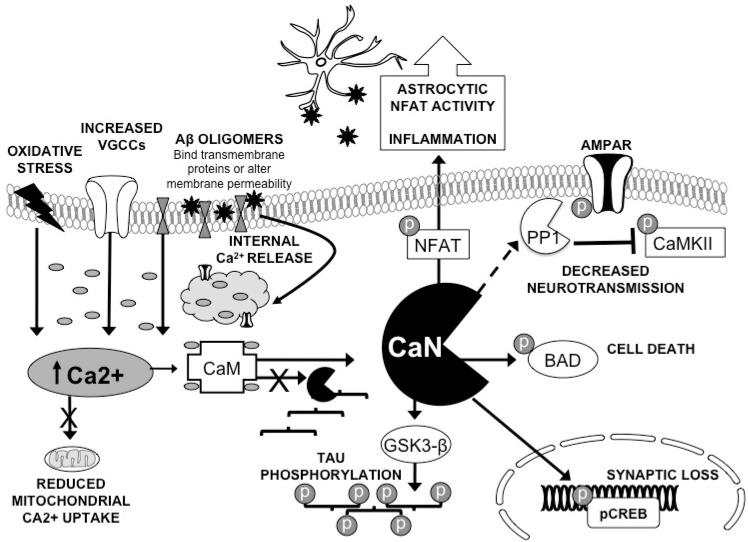
Calcineurin in the aging AD brain. The aged brain is susceptible to hyperactivation of CaN and downstream consequences due to its decreased ability to regulate intracellular Ca^2+^ levels. The additional insult of Aβ oligomers further disrupts synaptic homeostasis, resulting in a subtle, prolonged increase in calcium that facilitates the expression of LTD. Activation of CaN by CaM disrupts the phosphatases interaction with tau, possibily leading to tau hyperphosphorylation [92]. CaN also mediates the dephosphorylation of several cellular proteins: pCREB [8], pNFAT [81], p-PP1 [13, 53], p-GSK-3 [16], and pBAD [10, 11]. This could putatively explain four observations in AD models and pathogenesis; synaptic protein loss, neuroinflammation (neuronal and astrocytic), decreased neurotransmission, hyperphosphorylated tau, and cell death. Therefore, inhibition of CaN or the promotion of positive plasticity may serve as viable therapeutic strategies for combating early stage AD impairment.
